# Results of transoral laser microsurgery for supraglottic carcinoma in 277 patients

**DOI:** 10.1007/s00405-012-2327-6

**Published:** 2013-01-10

**Authors:** Martin Canis, Alexios Martin, Friedrich Ihler, Hendrik A. Wolff, Martina Kron, Christoph Matthias, Wolfgang Steiner

**Affiliations:** 1 Department of Otorhinolaryngology, Head and Neck Surgery, University of Göttingen, Robert-Koch-Str. 40, 37099 Göttingen, Germany; 2 Department of Audiology and Phoniatrics, University of Berlin, Berlin, Germany; 3 Department of Radiation Oncology, University of Göttingen, Göttingen, Germany; 4 Institute of Epidemiology and Medical Biometry, University of Ulm, Ulm, Germany

**Keywords:** Transoral laser microsurgery, Supraglottic laryngeal carcinoma oncologic result, Oncologic result, Functional outcome, Organ preservation

## Abstract

The objective of the study was to evaluate the oncological and functional results of transoral laser microsurgery (TLM) in patients with supraglottic laryngeal squamous cell carcinoma. Between June 1980 and December 2006, 277 patients with squamous cell supraglottic carcinoma of all stages were treated by primary carbon dioxide laser microsurgery. All treatments were performed with curative intention. The goal was the complete tumor removal with preservation of functionally important structures of the larynx. The administered treatment was exclusively TLM with or without selective or modified radical neck dissection in 215 cases (78 %); TLM with postoperative radiotherapy was performed in 62 cases (22 %). Data were analyzed using the Kaplan–Meier method. The median follow-up was 65 months. We achieved a 5-year local control rate of 85 % for pT1/pT2, 82 % for pT3, and 76 % for pT4. The 5-year overall, recurrence-free and disease-specific survival rates for stages I and II were 76, 81, and 92 %, for stages III and IVa 59, 65, and 81 %, respectively. With respect to local control and survival, these results are comparable with the results achieved by conventional partial and total resection of the larynx, while being superior to primary (chemo)radiotherapy. Transoral laser microsurgery results in a low morbidity, rapid recovery, and superior function compared with standard therapy.

## Introduction

It has been shown that supraglottic laryngectomy is an effective approach for supraglottic carcinoma in early stages, as is total laryngectomy for most advanced tumors. Radiotherapy is an accepted alternative to surgery in early stage disease, especially in the elderly and multimorbid patients with pulmonary dysfunction, because of significant morbidity and postoperative functional problems after classic partial supraglottic laryngectomy. Alonso [1] described horizontal supraglottic partial resection in 1947 and Vaughan [2] was the first to describe the use of the CO_2_ laser resection of supraglottic carcinomas in 1978, mainly as staging procedure. The first steps in the treatment of benign lesions and the early stages of malignant supraglottic lesions were discussed by Davis et al. [3] in 1983. This first series of 20 patients was followed by series of selected cases reported by Zeitels et al. [4] in 1990 and Davis et al. [5] in 1991. Steiner commenced transoral laser microsurgery (TLM) for carcinomas of the larynx in 1979. Since the early eighties he expanded the indications to all tumor categories of the upper aero-digestive tract [6–9]. Within the last 20 years, glottis-preserving TLM has gained importance in the surgical treatment of supraglottic carcinoma. At initial consideration, TLM seems to contradict classic rules of oncological surgery because tumors are divided and removed in several pieces [6, 10]. However, the hemostatic effect of the laser seals tissues, e.g., lymphatic microvessels without a higher risk of tumor spread compared with open approach [11]. Furthermore, due to the specific cutting properties of the CO_2_ laser a differentiation between healthy tissue and tumor is possible under the operating microscope by cutting through the tumor [10]; therefore, more healthy functional important structures can be preserved. TLM has several further advantages over conventional supraglottic laryngectomy, including avoidance of tracheotomy in most cases, faster and better recovery of swallowing function, lower incidences of aspiration or pneumonia, no pharyngocutaneous fistulae, shorter time of surgery, and shorter period of hospitalization.

The aim of this study was to assess the effectiveness of TLM in the treatment of supraglottic laryngeal carcinoma and to compare its oncological and functional results with those of open surgery and radio(chemo-)therapy. For this reason local control, organ preservation rate, stage related survival, regional and distant metastatic spread, and functional outcome were analyzed. In addition to primary site tumor resection, patients were optionally treated by unilateral or bilateral neck dissection (mainly selective neck dissection), and postoperative (chemo)radiotherapy, depending on tumor stage (mainly for advanced neck disease).

## Patients and methods

### Patients

Between June 1980 and December 2006, a total of 277 previously untreated patients with squamous cell carcinoma of the supraglottic larynx (T1-4, N0-2, M0) were treated with curative intent at the Departments for Otorhinolaryngology, Head and Neck Surgery of the University of Erlangen (1980–1985, surgery by the senior author) and Göttingen (8/1986–12/2006, surgery by 7 surgeons). Since 1986, a prospective database has been maintained of all patients undergoing TLM at the University of Göttingen Medical Center. Of these patients, 46 were females (16 %) and 231 were males (84 %) with a median age of 59 years (range 26–89 years). All surgical procedures were performed exclusively by a transoral approach; none of the patients underwent open surgery for primary treatment. In addition to the transoral microsurgical laser excision of the primary tumor, selective or modified radical neck dissections and/or adjuvant radiotherapy were performed, depending on the extent of laryngeal and neck disease.

Two hundred and one patients from Göttingen with supraglottic carcinoma, 173 (86 %) males and 28 (14 %) females, were excluded from the study with the following exclusion criteria: in 42 cases the patients presented at our department with a recurrence of an already treated carcinoma; 71 patients were diagnosed with a simultaneous second primary, 7 with simultaneous distant metastases; 19 patients underwent primary radiotherapy; a further 38 patients were treated by primary total laryngectomy due to very advanced disease with no possibility for organ preserving TLM. Eight patients were excluded because of advanced neck disease (N3). In seven further cases, the intention of treatment was palliative, two patients because of histological findings (non squamous cell carcinoma), and seven due to other reasons; 44 (22 %) of these excluded patients had stage I and II disease, 157 patients (78 %) belonged to stage III and IV.

### Preoperative examination

Examination prior to all treatment consisted routinely of magnifying rigid or flexible laryngoscopy, phoniatric analysis and documentation [10], palpation and, since 1989, ultrasonography of the neck. Fine needle biopsies of the neck nodes, if necessary under ultrasonographic control, were performed in selected cases. Computed tomography (CT) was not routinely employed in early stages. In cases of suspected advanced disease, especially neck infiltration, CT or MRI scans were performed to verify invasion of the neck unless the patient presented with satisfactory imaging performed at the referring hospital.

Panendoscopy was performed under general anesthesia at the beginning of the surgical procedure for laser biopsy if the carcinoma was not yet proved by histology and with the intention to exclude any second primary tumor in the aero- and upper digestive tract. Distant metastases were excluded by means of radiography of the chest and ultrasonography of the abdomen. Additional pulmonary function tests, such as vital capacity and forced expiratory volume in 1 s, were done only in patients with a history of chronic obstructive pulmonary disease.

### Operative technique

The surgical procedures were always performed under general anesthesia. Orotracheal intubation was performed by the surgeon under endoscopic control. In most cases, a distending laryngopharyngoscope (like Steiner’s bi-valved adjustable laryngopharyngoscope, Karl Storz, Tuttlingen, Germany) offered decisive advantages over conventional closed laryngoscopes in exposing the supraglottic tumor when using the transoral approach. During exposition of the tumor, the lower blade of the laryngoscope displaced the endotracheal tube, whereas the upper blade was placed in the vallecula. This positioning of the laryngoscope usually had to be modified several times during operation. This was necessary, as good exposure and visualization are prerequisites of a safe and successful, function preserving resection. In order to remove laser plume, the laryngoscopes are equipped with integrated suction tubes. In some cases, small closed laser laryngoscopes were used for a better exposure of the anterior glottis and subglottis.

We used a CO_2_ laser system (40c, Lumenis, Dreieich, Germany) to perform the surgical procedures. This laser system was equipped with a micromanipulator attached to the operating microscope (OPMI Vario/S88, Zeiss, Göttingen, Germany). The focus diameter of the micromanipulator was 0.5 and 0.25 mm, respectively; and approximately 2,080 to 3,900 W/cm^2^ of laser energy was transmitted. The amount of laser power used varied from 3 W for excisions of the vocal fold lesions to 15 W when cutting through a large tumor mass. This administered laser power was able to coagulate vessels with a maximum diameter of 1 mm. When larger vessels of the superior laryngeal vasculature were opened during surgery, vessels were clearly defined and double clipped.

Small carcinomas of the free border of the epiglottis, or the aryepiglottic fold, were resected in one piece as an excisional biopsy. More extended tumors were excised stepwise in several portions. This unconventional surgical technique was introduced by Steiner in the early 1980s [6, 10] and is justified, because the lymphatic vessels of the wound margins are sealed immediately, as shown by Werner [11]. A subsequent increase of late neck or distant metastases on long-term follow-up has not been found as demonstrated by Caballero et al. [12].

The first step was usually an incision in the vallecula followed by a sagittal splitting of the epiglottis in the midline [10]. Then, both parts of the suprahyoid epiglottis were removed followed by the two infrahyoid parts. Massive tumor invasion of the pre-epiglottic compartment could be seen under the microscope as soon as the infrahyoid epiglottis was divided and removed with the laser. Because of the possibility of microscopic invasion, the pre-epiglottic fat was always partially or totally resected in cases of infrahyoid epiglottic involvement. Depending on the extension of the tumor, one or both false vocal cords, portions of the vocal folds and, if necessary, the pre-epiglottic space were partly or completely dissected.

If the histopathological analysis of the resected specimens (frozen sections) showed a positive resection margin, an additional resection was carried out to obtain negative margins until an adequate distance between the margin of the tumor and the resection borders was achieved.

If the thyroid cartilage was exposed extensively, or partially removed during resection of the tumor, we administered perioperative penicillin or clindamycin to avoid a possible perichondritis.

### Treatment of the primary tumor

All 277 patients were classified according to the current UICC/AJCC classification [13]. 24 patients (9 %) belonged to stage I, 75 (27 %) stage II, 88 (32 %) stage III, and 90 (32 %) stage IV. Postoperative TN categories are given in Table 1.

**Table 1:** Distribution of the pT and pN categories of all 277 patients

	pT1	%	pT2	%	pT3	%	pT4	%	Total	%
N0/pN0	22	7.9	68	24.5	55	19.9	22	7.9	167	60.3
N1	3	1.1	8	2.9	16	5.8	10	3.6	37	13.4
N2	1	0.4	16	5.8	33	11.9	23	8.3	73	26.4
	26	9.4	92	33.2	104	37.5	55	19.9	277	100.0

Sixty one patients (22 %) were exclusively treated with TLM, 155 (56 %) with laser surgery and neck dissection, 59 (21 %) with laser surgery, neck dissection, and adjuvant (chemo)radiotherapy, and 2 patients (1 %) with laser surgery and adjuvant (chemo)radiotherapy. Median follow-up was 65.63 months (range 1–235 months) (Table 2).

**Table 2:** pN categories of all 214 patients after neck dissection

pN	*N*	%
0	127	59
1	32	15
2a	3	2
2b	31	14
2c	21	10
	214	100

### Diagnosis and treatment of the neck

Preoperatively all patients were assessed for lymph node metastasis by ultrasonography, and/or CT/MRI. Follow-up consisted of ultrasound and endoscopic examination every 2–3 months in the first 2 years, then every 6 months.

If the patient presented with advanced primary disease or the tumor infiltration depth was >4 mm [13], or preoperative imaging revealed suspicious lymph nodes, a uni- or bilateral selective neck dissection was performed. Patients underwent bilateral neck dissection if imaging revealed suspicious lymph nodes bilaterally, or if primary tumor showed an advanced stage with midline localization, or if suspicious lymph nodes were seen only on one side, but no adjuvant (chemo)radiotherapy was planned.

Neck dissection was performed in 214 patients (77 %), 147 patients (69 %) were treated bilaterally and 67 (31 %) ipsilaterally to the primary tumor site, making a total of 361 neck dissections. In all 214 patients, a level II/III selective neck dissection was performed. In addition, level I was dissected in eight patients, level IV in six patients, and level V in six patients. A bilateral modified radical neck dissection (level I-V) was done in three cases.

### Adjuvant (chemo)radiotherapy

Adjuvant (chemo)radiotherapy was mainly performed in cases of advanced neck disease (N2a/b/c) or when the histopathological examination revealed extracapsular spread and/or lymphatic micrometastases. From 11/1980-12/1994 (29 patients), the (chemo)radiotherapy schedule was composed of two fractions per day, separated by 6 h intervals. Each fraction consisted of 210 cGy (1.25 MV ^60^CO) preceded by a dose of 50 mg/m^2^ carboplatin i.v. daily. A total radiation dose of 5,670 cGy was applied to the neck and the primary tumor over 6 weeks as split-course regimen. A break of 2 weeks was planned between the two last weeks of treatment. Treatment was given 4 days a week. Cisplatinum-based chemotherapy was given concomitantly in nine cases.

From 01/1995–12/2004 (29 patients), normofractionated radiotherapy (2 Gy/d, 5 times/week) was delivered as follows: parallel, opposed lateral portals were applied, matched to a single anterior portal encompassing the primary tumor and associated nodal drainage sites up to a maximum dose of 50 Gy. Finally, a 3D-conformal-external-beam radiotherapy technique was used to boost the total dose to 60 Gy, including the primary tumor and involved lymph nodes. The spinal cord was limited to a maximum of 45 Gy. Concomitant chemotherapy was applied in three cases.

From 12/2004–12/2006 (3 patients), normofractionated (2 Gy/d, 5 times/week) 3D-conformal-external-beam radiotherapy was given with concomitant Cisplatinum-based chemotherapy in two cases. The primary tumor and involved lymph nodes and potential drainage sites on both sides of the neck, including the supraclavicular region, were covered with 50 Gy in a first phase followed by a boost up to a total dose of 64 Gy, including the primary tumor and involved lymph nodes. The spinal cord was limited to a maximum of 45 Gy.

### Statistical methods

All survival rates were calculated using the Kaplan–Meier method. The assessed endpoints were overall survival, recurrence-free survival, disease-specific survival, local and loco-regional control, distant metastases, and second primaries.

Overall survival was calculated on deaths from all possible causes, disease-specific survival on deaths from supraglottic cancer. For calculating recurrence-free survival, intercurrent deaths, and deaths due to secondary primary tumors, as well as patients alive without recurrences were regarded as censored observations. Events included local and regional recurrences; distant, recurrent, and late metastases; and deaths due to disease. For the calculation of local control rate, only local recurrences were considered as events while patients alive without local recurrence or death regardless of reason were counted as censored observations. The definition of local recurrence included carcinoma in situ as well as a carcinoma occurring after completion of primary treatment. Survival times were calculated from the day of surgery to the date of the occurrence of the event or the date of the last follow-up.

## Results

### Oncologic results

**Fig. 1:**
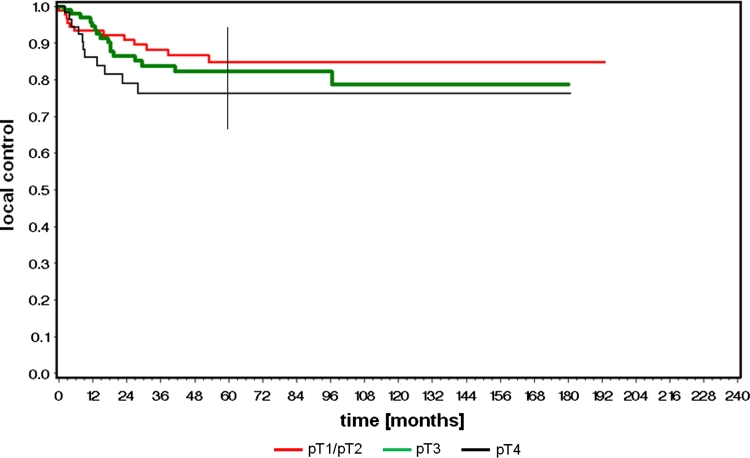
Local control rate for all patients related to T-category

Figure 1 depicts the local control rate after TLM for all patients related to T category. Due to small numbers in the pT1 group (*n* = 26), pT1 and pT2 patients were analyzed together. We achieved a 5-year local control rate of 85 % for pT1/pT2, 82 % for pT3, and 76 % for pT4.

Local or loco-regional recurrences were observed in 1 of 26 patients with pT1 tumor (4 %), in 14 of 92 patients with pT2 tumor (15 %), in 21 of 104 patients with pT3 tumor (20 %), and in 12 of 55 patients with pT4 tumor (22 %). Thus, in 48 patients (17 %) local or loco-regional recurrence occurred, although the analyzed histological margins of the primary tumor resection including re-resection were free of tumor in all cases. All rpT categories of the first local or loco-regional recurrence are given in Table 3. Tables 4 and 5 give patterns of TN- and stage-related first failures after initial therapy.

**Table 3:** rpT categories of all 48 first local and loco-regional recurrences

	rT1	rT2	rT3	rT4	Total	%
pT1 (*n* = 26)	0	0	1	0	1	2
pT2 (*n* = 92)	4	4	1	5	14	29
pT3 (*n* = 104)	5	3	0	13	21	44
pT4 (*n* = 55)	1	1	2	8	12	25

**Table 4:** Treatment of the first failures (TN) after initial therapy depending on T-category

pT	*N*					Σ
		Loc. rec.	Loco-reg. rec.	Late metastasis	Rec. metastasis	
1	26	1 (4 %)	0	1 (4 %)	1 (4 %)	3 (12 %)
2	92	12 (13 %)	2 (2 %)	3 (3 %)	0	17 (18 %)
3	104	16 (14 %)	6 (6 %)	6 (6 %)	5 (5 %)	33 (31 %)
4	55	11 (20 %)	1 (2 %)	0	3 (5 %)	15 (27 %)
	277	39 (14 %)	9 (3 %)	10 (4 %)	9 (3 %)	68 (24 %)

**Table 5:** Treatment of the first failures (TN) after initial therapy depending on stage

Stage	*N*					Σ
		Loc. rec.	Loco-reg. rec.	Late metastasis	Rec. metastasis	
I	24	1 (4 %)	0	1 (4 %)	0	2 (8 %)
II	75	10 (13 %)	2 (1 %)	3 (4 %)	0	15 (19 %)
III	88	16 (17 %)	4 (5 %)	5 (6 %)	2 (2 %)	27 (30 %)
IVa	90	13 (14 %)	3 (3 %)	1 (1 %)	7 (8 %)	24 (27 %)
	277	39 (14 %)	9 (3 %)	10 (4 %)	9 (3 %)	68 (24 %)

Salvage therapy after the first failure (*n* = 68) is given in Table 6. In total 19 patients presented with a second failure: 12 with local recurrence, 2 with loco-regional recurrence, 1 with second late neck metastases, and 4 with second recurrent neck metastases.

**Table 6:** Salvage therapy after the first failure for all 68 patients

Salvage therapy after first event	
Laser	18
Laser + ND	2
Laser + R(C)T	4
Laser + ND + R(C)T	1
ND	4
ND + R(C)T	9
Laryngectomy	8
Laryngectomy + R(C)T	9
R(C)T	5
Palliative treatment	6
Lost to follow-up	2
	68

Organ preservation could be achieved in 26 of 26 pT1 cases (100 %), in 88 of 92 pT2 cases (96 %), in 97 of 104 pT3 cases (93 %), and in 46 of 55 pT4 cases (84 %).

Second primary tumors were observed in 55 patients (20 %). In 19 of these patients, the second primary tumor was localized in the head and neck region, in 36 patients the second primary occurred in the lung (*n* = 19), GI-tract (*n* = 10), prostate (*n* = 2), and others (*n* = 5). The second primary tumors were diagnosed between 8 and 153 months (mean 76 months) after laser resection.

One hundred thirty-eight patients (49.8 %) are living without any evidence for tumor recurrence, 3 patients (1.1 %) are living with tumor, 39 patients (14.1 %) died from TNM-related reasons, 62 patients (22.4 %) from intercurrent diseases, and 34 (12.3 %) from second primary tumors. One patient (0.3 %) was lost to follow up.

**Fig. 2:**
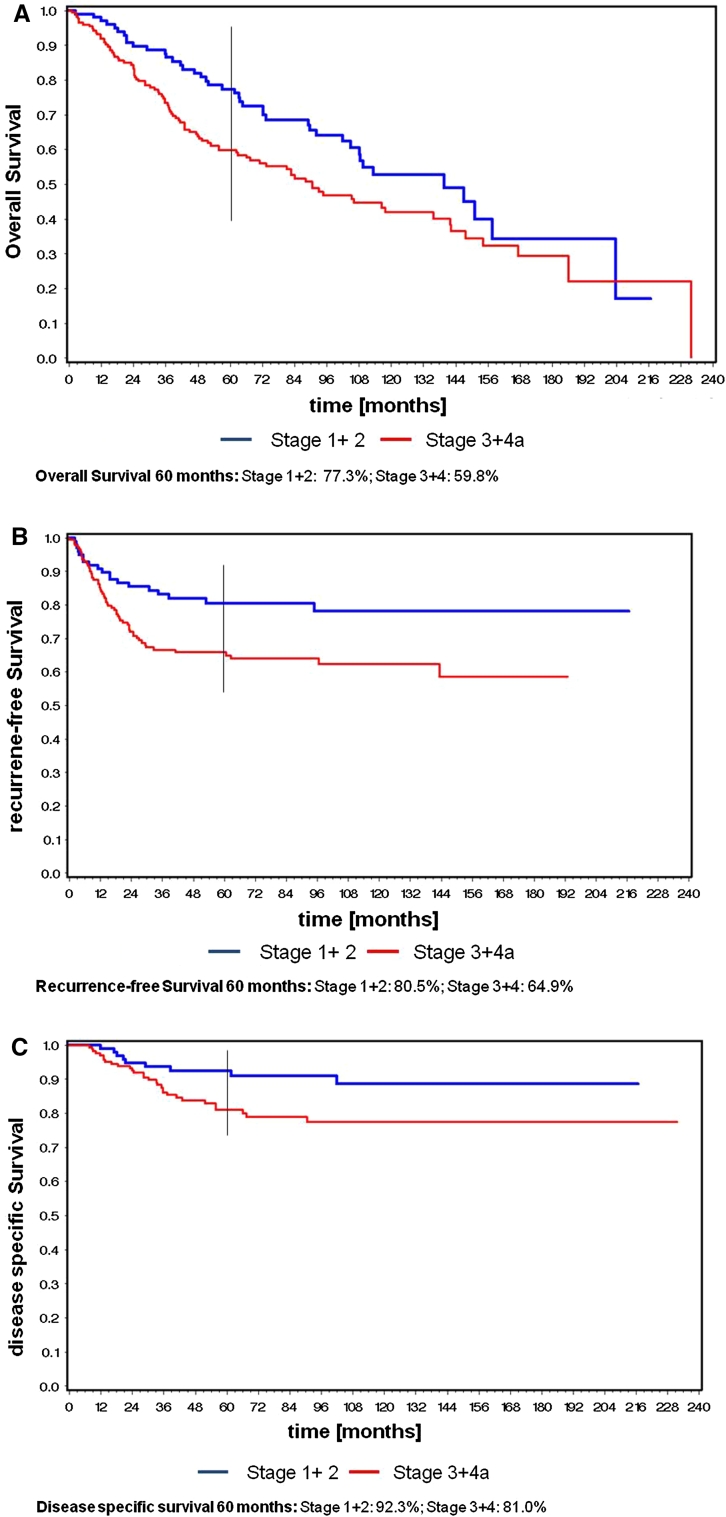
**a** Overall survival for stages I and II versus III and IVa. **b** Recurrence-free survival for stages I and II versus III and IVa. **c** Disease-specific survival for stages I and II versus III and IVa

Five-year overall, recurrence-free, and disease-specific survival rates for stage I and II were 77, 81, and 92 %; for stage III and IVa 59, 65, and 81 % (Fig. 2a–c).

Histopathologically positive lymph nodes were found in 87 of 214 patients (41 %), 21 (10 %) patients showed bilateral neck disease. Contralateral without ipsilateral neck disease was not seen. On average 25 lymph nodes (SD 6.32; range 5–42) were removed. Adjuvant radiotherapy was performed in 62 cases (22 %) combined with chemotherapy in 19 cases (5 %).

There was no statistically significant difference in loco-regional recurrence rate, disease-specific survival, or organ preservation between patients who did or did not undergo adjuvant radiotherapy when all patients were compared. Although at first surprising, this is due to selection bias in this retrospectively analyzed patient cohort: patients who underwent radiotherapy had on average more extensive disease, making unfavorable outcome more likely; while 97 of the 215 patients without radiotherapy suffered from stage I or II disease, stage IVa was predominant with 45 of the 62 patients receiving radiotherapy.

### Complications and functional results

Nasogastric feeding tubes were needed postoperatively by 214 patients (77 %) with a median duration of 7 days and a range of 1–114 days. 13 (5 %) patients needed temporary gastrostomy tubes due to persistent aspiration. Following gradual improvement these were removed between 2 months and 42 months later. Five patients (2 %) needed permanent gastrostomy tubes. In all five patients, tumor resection had to be extended including parts of the tongue base and the hypopharynx. Nasogastric feeding tubes and gastrostomy tubes were removed only when the patients were able to eat a normal diet without any clinical and radiologic signs of aspiration.

Three patients (1 %), one of them with a pT3 cancer and two with a pT4 tumor, had to undergo total laryngectomy because of persisting aspiration. Pneumonia due to aspiration was observed in five cases (2 %).

Twenty-six patients (9 %) experienced postoperative bleeding, 22 (85 %) in the larynx requiring coagulation, and/or clipping in the operating room under general anesthesia. Four cases developed cervical hematoma after neck dissection and had to be managed in the operating room. No patient died due to bleeding, aspiration and/or airway compromise. No patient needed tracheotomy intraoperatively. However, postoperatively 1 patient required temporary tracheotomy due to bleeding, 11 patients (4 %) due to swelling of the larynx with dyspnoea. One underwent microlaryngoscopy to treat vestibular stenosis successfully.

## Discussion

Standard treatment regimes for supraglottic larynx carcinoma worldwide involve the options of partial or total laryngectomy and/or (chemo)radiotherapy. However, transoral laser microsurgical procedures, especially for advanced stage supraglottic carcinoma, are still controversial. In the last two decades, there have been several publications reporting successful laser microsurgical treatments in Europe [7, 14–16] and in the United States [5, 17, 18]. Successful treatment can be measured with the parameters of primary control, percentage of organ preservation, neck control, and survival. Nonetheless, any discussion of the oncologic outcomes is challenging, since evaluation of the evidence supporting the effectiveness of one treatment over the other is complicated by different stages, outcome measures, use of statistics, and investigations of different laryngeal sites (supraglottic vs. glottic vs. all sites).

### Local and loco-regional control

In our series patients with cancer of the supraglottic larynx and all T categories were managed by transoral laser surgery with or without selective neck dissection and with or without (chemo)radiotherapy. Five-year overall local control rate for stage I and stage II was 87.5 % and for stage III and IV was 81.1 %.

Local control rates of conventional supraglottic laryngectomy range from 90–100 % for T1, 80–97 % for T2, and 71–94 % for T4 carcinomas. In a retrospective review of 202 patients with supraglottic squamous cell carcinoma of all stages, Lutz et al. [19] achieved high local control rates with primary failures in 2 % of patients (3 of 130) following total laryngectomy and in 1 patient of 72 (1 %) treated by conventional supraglottic partial resection. Bocca et al. [20] reported a 2-year local recurrence rate of 16.5 % (stage I and II) and 21.5 % (stage III and IV) for open surgery. The vast majority in his series showed pT2 cancer (70 %), and additional 13 % pT1 and 17 % pT3. So the results were achieved with 83 % of the patients presenting with pT1 or pT2 tumor. In comparison, our patients showed pT1 or pT2 tumor in 42 % and pT3 or pT4 tumor in almost 48 % and we achieved a 2-year local control rate of 93 % for pT1/2, 93 % for pT3, and 84 % for pT4 tumors.

Primary radiotherapy can achieve local control rates of 77–100 % in supraglottic T1 cancers and 62–83 % in T2 cancers [21–25]. For supraglottic T3 carcinomas local control rates of 50–76 % are published [26–28]. Mendenhall et al. [23] reported 5-year local control rates after radiotherapy of 83 % for T2 and 68 % for T3 tumors. Our corresponding results were 85 and 82 %. While results are comparable in smaller T2 tumors, transoral laser surgery has a clear advantage in pT3 cases. In a series of 274 patients treated for supraglottic carcinoma by radiotherapy alone or radiotherapy with neck dissection, Hinerman et al. [26] reported an actuarial probability of local control of 100 % for T1, 86 % for T2, 62 % for T3, and 61 % for T4.

Using TLM, Ambrosch et al. [14] achieved a local control of 89 % in early T1-2 supraglottic carcinoma. Iro et al. [29] found stage-dependent 5-year local control rates of 86.1 % for stage I, 74.6 % for stage II, 75.4 % for stage III, and 78.4 % for stage IV. Grant et al. [30] investigated results of 38 patients undergoing TLM for carcinoma of the supraglottic larynx of all T categories. Twenty-six patients (68 %) underwent neck dissections. Thirteen patients (34 %) received adjuvant radiotherapy. The 2-year Kaplan–Meier estimates for local control were 97 %. In our study, we achieved 91 % for early disease and 85 % for advanced disease. Beside small groups the slightly better results presented by Grant et al. [30] may be explained by the fact, that 42 % of the patients presented with pT3/4 tumors, whereas in our series 64 % of the patients showed advanced disease.

Davis et al. [31] reported a local control of 97 % for 48 T2 tumors using TLM in combination with adjuvant (chemo)radiotherapy in 38 patients, which is in line with our results and data presented by Eckel [15] and Zeitels et al. [17].

Organ preservation in our study could be achieved in 26 of 26 pT1 cases (100 %), in 88 of 92 pT2 cases (96 %), in 97 of 104 pT3 cases (93 %), and in 46 of 55 pT4 cases (84 %). Overall, this results in larynx preservation of 93 %. In contrast to these results, Johansen et al. [32] report about salvage laryngectomy in 33 of 117 patients in T1 supraglottic carcinomas after primary radiotherapy. This translates into organ preservation of less than 72 % in T1 cancer. Hinerman et al. [26] treated patients with radiotherapy for supraglottic T3 carcinoma and were able to preserve the larynx in 68 %, Nakfoor et al. in 72 % of the cases [27].

### Neck control

Neck failure is generally seen as a major problem in the treatment of supraglottic carcinoma. Whether or not to surgically treat both sides of the neck routinely is still a controversy [19, 20, 33, 34]. Selective neck dissection was performed in 214 patients (77 %), in 147 patients (69 %) bilaterally and in 67 (31 %) homolaterally to the tumor site. A bilateral modified radical neck dissection (level I–V) was done in 3 cases (1 %). Of the ten patients who developed late neck metastases, six had been staged cN0 and four pN0. Nine patients developed recurrent neck metastasis after staging pN+ at the time of primary treatment.

Myers and Alvi [35] observed a total of 14 % neck recurrences in a series of 103 patients treated by supraglottic laryngectomy (32 %) or total laryngectomy (68 %) with unilateral neck dissections performed in 29 %, bilateral in 67 %, and without neck dissection in 4 %. The use of adjuvant radiotherapy has been based on the presence of extracapsular spread.

### Survival

When discussing our results, it has to be mentioned that our patients included a high number of stage III (32 %) and stage IV (32 %) tumors. In our series we measured 5-year overall, recurrence-free and disease-specific survival rates for stage I and II of 77, 81, and 92 %; for stage III and IVa of 59, 65, and 81 %.

In a large review of the National Cancer Data Base, Hoffman et al. [36] investigated data of 158,426 patients with laryngeal cancer. The authors found that survival has decreased among patients with laryngeal cancer during the past two decades in the United States. During this time there has been an increase in the nonsurgical treatment of laryngeal cancer. Advanced T3 laryngeal cancer 5-year observed and relative survival showed the best outcome for patients whose initial management was surgery, either alone or combined with irradiation. Observed survival rates were 52.9 % for surgery alone, 33.1 % for irradiation, 55.4 for surgery and adjuvant irradiation, and 50.7 % for (chemo)radiotherapy.

Oncologic results of transcervical approaches have been presented previously in several studies [37–39]. For transcervical supraglottic laryngectomy, 5-year survival rates range between 68 and 89 % (all stages); for total laryngectomy between 55 and 80 %. Lutz et al. [19] investigated the outcome of 202 patients with supraglottic carcinoma of the larynx after conventional surgery. The authors state a 2-year survival rate of 67 % for stage I, 82 % for stage II, 84 % for stage III, and 55 % for stage IV. Our corresponding 2-year overall survival results were 90 % for stage I and II tumors and 83 % for stages II and IVa. The results of Lutz et al. include 130 patients (60 %) who underwent primary total laryngectomy whereas in our series only 20 of 277 patients (7 %) needed secondary total laryngectomy as a salvage procedure.

In a large retrospective study (901 patients), Scola et al. [33] reported a 5-year uncorrected actuarial survival rate of 84, 81, 76, and 55 % for stages I, II, III, and IV. All 12 stage I cases were treated by conventional or extended supraglottic laryngectomy. Again, there were no significant differences in survival compared with our organ and function preserving results.

Spaulding et al. [25] reported a series of 162 patients with supraglottic cancer treated with radiotherapy and surgical salvage, preoperative radiotherapy and surgery, total laryngectomy and postoperative radiotherapy, and partial laryngectomy with postoperative radiotherapy. The 3-year determinant survival rate is stated as 65 % for T1 cancer, 81 % for T2, 57 % for T3, and 61 % for T4. Our corresponding 3-year overall survival results were 86 % for early disease (pT1/2) and 73 % for advanced disease (pT3/4) and thus compare favorably.

Primary (chemo)radiotherapy can achieve a corrected 5-year survival rate of 70 % as presented by Sykes et al. [28] 331 patients with clinically node-negative supraglottic carcinoma of the larynx were treated with radiotherapy. Examined by T-category, corrected survivals were 83, 78, 53, and 61 % for T1, T2, T3, and T4 tumors respectively. Mendenhall et al. [40] reported 129 patients with supraglottic cancer (all stages) which were treated by radiotherapy alone or followed by neck dissection. The authors observed an absolute 5-year survival rate of 58 % for all stages. Nakfoor et al. [27] treated 190 patients with carcinoma of the supraglottic larynx with primary (chemo)radiotherapy. For T1, T2, T3, and T4 tumors, relapse-free survival rates were 78, 82, 64, and 40 %.

The first paper of the Göttingen study group was published in 1998 and presented good results for early supraglottic laryngeal carcinoma (pT1/pT2) [14]. 48 patients with supraglottic carcinoma were treated almost exclusively (96 %) by TLM. The 5-year recurrence-free rate and 5-year overall survival rate were 83 and 76 %, respectively. In our large series of 277 patients, the 5-year recurrence-free and 5-year overall survival rates were 81 and 77 %, respectively. These data are in line with the good results presented by Ambrosch et al. [14].

Iro et al. [29] reviewed the medical records of 141 patients with supraglottic carcinomas undergoing transoral laser surgery, facultatively in combination with neck dissection or radiotherapy. Five-year recurrence-free survival rates were 65.7 %; stage I, 85.0 %; stage II, 62.6 %; stage III, 74.2 %; and stage IV, 45.3 %. The local and regional recurrence rates were 16.3 and 9.9 %, respectively. Vilaseca et al. [18] investigated 147 patients with T3 glottic and supraglottic laryngeal carcinoma and observed a 5-year disease-specific survival rate of 61.8 % for supraglottic carcinoma.

In our series we measured 5-year overall, recurrence-free and disease-specific survival rates for stage I and II of 77, 81, and 92 %; for stage III and IVa of 59, 65, and 81 % which are good results corroborating the relevance of TLM as a valuable option for the treatment of supraglottic laryngeal cancer.

### Complications

Twenty-six patients (9 %) experienced postoperative bleeding, 22 (85 %) in the larynx requiring coagulation and/or clipping in the operating room under general anesthesia. This is slightly higher than the occurrence of postoperative bleeding reported after open supraglottic laryngectomy, which varies between 1.6 and 5 %, according to Herranz-Gonzalez et al. [38].

In their large series of 901 patients, Scola et al. [33] state an incidence of 3.8 % for pneumonia and 4.5 % for fistula. We observed five pneumonias (2 %) and no fistulas in our own study. Roh et al. [41] show the incidence of aspiration pneumonia after TLM of 11.5 % and after transcervical approach up to 40 %. Lower complication rates after TLM may be attributed to the preservation of healthy tissue and functionally important structures.

In contrast to the 5 % incidence of vestibular stenosis after open supraglottic laryngectomy, as stated by Staffieri [42], or 2.5 % observed by Herranz-Gonzalez et al. [38], we saw only 1 (0.4 %) stenosis after extensive laser resection of the supraglottis and paraglottic space bilaterally. This low incidence may be attributed to the lack of wound infection and perichondritis and the preservation of the framework of the larynx, contrary to the findings of Laccourreye et al. [43], who observed significant complications after partial laryngectomy and radiotherapy. Although their patients were highly selected (exclusion of patients with postoperative complications before adjuvant radiotherapy), there were five cases of laryngeal radionecrosis (5.5 %), four laryngeal stenoses (4.4 %), two stenoses of the esophageal inlet (2.2 %), and three pneumonias (3.3 %). More severe complications caused by radiotherapy were reported by Mendenhall et al. [23] who observed a carotid blowout in one patient, one partial spinal transsection, three laryngectomies and one permanent tracheotomy because of chondronecrosis and another two permanent tracheotomies due to laryngeal edema.

### Functional results

The 4 % rate of temporary tracheotomy compares favorably with reports of conventional surgery by Robbins et al. [39] who reported an incidence of permanent tracheotomies of 6 %, the 6 % failed decannulations observed by Herranz-Gonzalez et al. [38] and the up to 15 % of delayed decannulation or late recanulation reported by Krespi and Kheterpal [44] in a literature review. Scola et al. [33] observed failed decannulation in 9 % and delayed decannulation (more than 1 month postoperatively) in 38 %. Spirano et al. [45] observed a median time to decannulation of 4.9 months and a persistent aspiration in 9 %. Kollisch et al. [46] compared functional results following transcervical supraglottic laryngectomy and transoral laser resection. Concerning swallowing function, tracheotomy rate, and aspiration pneumonia, TLM was superior compared with the transcervical approach. Tracheotomy rates following TLM for supraglottic carcinoma range between 0 and 32 %. In our series 4 % of patients received temporary tracheotomy due to aspiration or bleeding. These results are in favor of laser microsurgery since with the open approach temporary tracheotomy is necessary in almost every patient.

Seventy-four percent of our patients had their feeding tubes removed within the first 14 days. An additional 18 % were weaned from tube feeding after a total of 30 days. Prolonged tube feeding (31–90 days) was necessary in 6 % of our patients. This is comparable to delayed swallowing in 5.5 % of open supraglottic laryngectomy as reported by Bocca [20]. Five of our patients (2 %) needed permanent gastrostomy and a total of three patients had to undergo total laryngectomy because of aspiration. Taking into account that the majority of our patients had stage III and IV disease we think that the result of 3 % (8 patients) with a failed swallowing function restoration after TLM is acceptable.

Bernal-Sprekelsen et al. [47] made a valuable contribution on aspiration rehabilitation specifically in patients after TLM of 210 patients presenting with malignant tumors of the hypopharynx and larynx. Endoscopic resections included T2 to T4 tumors, and nasogastric feeding tube was used in 23.2 % of small tumors and in 63 % of locally advanced tumors. Twelve patients (5.7 %) had postoperative pneumonia and 59 (28.1 %) had temporary aspiration that correlated with location and locally advanced tumors. The authors concluded that endoscopic resection of laryngeal and hypopharyngeal tumors is associated with good recovery of deglutition.

As a comparison, Schwaab et al. [48] report a rate of 9 % total laryngectomies (13 patients) in a series of 146 patients treated by supracricoid partial laryngectomy with cricohyoidopexy, because of severe postoperative aspiration. Logemann et al. [49] identified the impaired larynx sphincter function and the movement of the tongue base, as the two critical factors for recovery of swallowing. The extent of aspiration depends on the achievement of these two functions and on the age of the patient. In addition to that, preservation of the sensory function seems to be important for restituting swallowing function, according to Flores et al. [50]. It seems therefore feasible to preserve as much hyoid bone, strap muscle, and parts of the superior laryngeal nerve branches as possible.

## Conclusion

Primary TLM in combination with or without neck dissection and (chemo)radiotherapy offers good oncologic and functional results to patients with supraglottic carcinoma. This approach has considerable advantages including lower morbidity, shorter duration of treatment, and better patient acceptability. TLM combines local disease control (by an intraoperative differentiation between tumor and healthy tissue allowing oncologically radical tumor resection) while preserving as much healthy and functionally important tissue as possible. Furthermore, the accurate indication for selective neck dissection and adjuvant therapy is possible following intraoperative and postoperative histological assessment of tumor depth and invasion. The technique also does allow de-escalation of treatment for those cases deemed to be satisfactorily treated by TLM alone, plus/minus selective neck dissections, a decision based on the full pathological assessment.

The results achieved by transoral laser microsurgical resection are at least as good compared with those of open supraglottic or total laryngectomy with respect to local control and survival, for both early and advanced stages. The results after TLM are slightly better than the results reported with primary radiotherapy for early disease and clearly superior regarding advanced disease.

The relatively low postoperative morbidity and good functional results are achieved by performing resection tailored to the real tumor spread. It is possible to preserve functional important structures and thus to permit early and successful swallowing rehabilitation and to avoid primary tracheotomy in most cases. Further benefits are the possibility to integrate transoral laser surgery into any therapeutic concept and the lack of necessary reconstructive surgery. These results are satisfactory and encourage us to continue and recommend TLM for supraglottic laryngeal carcinoma. As it is the data of only one institution, it is hoped that these results should be validated by prospective multicenter studies.
